# sciReptor: analysis of single-cell level immunoglobulin repertoires

**DOI:** 10.1186/s12859-016-0920-1

**Published:** 2016-02-04

**Authors:** Katharina Imkeller, Peter F. Arndt, Hedda Wardemann, Christian E. Busse

**Affiliations:** Division of B Cell Immunology, German Cancer Research Center, Feld 280, Heidelberg, 69120 Germany; Department for Computational Molecular Biology, Max Planck Institute for Molecular Genetics, Ihnestrasse 63-73, Berlin, 14195 Germany

**Keywords:** Single cell, Antigen receptor repertoire, Immunoglobulin, B cell repertoire

## Abstract

**Background:**

The sequencing of immunoglobulin (Ig) transcripts from single B cells yields essential information about Ig heavy:light chain pairing, which is lost in conventional bulk sequencing experiments. The previously limited throughput of single-cell approaches has recently been overcome by the introduction of multiple next-generation sequencing (NGS)-based platforms. Furthermore, single-cell techniques allow the assignment of additional data types (e.g. cell surface marker expression), which are crucial for biological interpretation. However, the currently available computational tools are not designed to handle single-cell data and do not provide integral solutions for linking of sequence data to other biological data.

**Results:**

Here we introduce sciReptor, a flexible toolkit for the processing and analysis of antigen receptor repertoire sequencing data at single-cell level. The software combines bioinformatics tools for immunoglobulin sequence annotation with a relational database, where raw data and analysis results are stored and linked. sciReptor supports attribution of additional data categories such as cell surface marker expression or immunological metadata. Furthermore, it comprises a quality control module as well as basic repertoire visualization tools.

**Conclusion:**

sciReptor is a flexible framework for standardized sequence analysis of antigen receptor repertoires on single-cell level. The relational database allows easy data sharing and downstream analyses as well as immediate comparisons between different data sets.

**Electronic supplementary material:**

The online version of this article (doi:10.1186/s12859-016-0920-1) contains supplementary material, which is available to authorized users.

## Background

The assessment of immunoglobulin (Ig) heavy and light chain sequences is essential to investigate the cellular mechanisms underlying humoral immunity. This applies to the accurate quantification of repertoire diversity or hypermutation, maturation and differentiation dynamics of B cells during an immune response. Typically, this information is obtained by next-generation sequencing (NGS) of transcripts isolated from bulk B cell populations. The inherent drawback of this strategy is the loss of information regarding Ig heavy:light chain pairing, which is a critical determinant of antibody reactivity.

Approaches sequencing individually isolated cells naturally preserve this information. Moreover, single-cell resolution facilitates the integration of antigen receptor repertoire (ARR) information with various other data types, e.g. surface marker phenotypes as determined by flow cytometry (FC). Strategies of this type have been successfully applied in the investigation of numerous types of infections or auto-immune diseases. In the case of influenza vaccination e.g. the combination of whole antibody sequence data together with single-cell phenotyping and affinity measurements of monoclonal antibodies has provided insights into the characteristics of memory recall, affinity maturation and selection as well as epitope specificity of the B cell response [1–4].

Historically the assessment of single Ig sequences has been performed using Sanger sequencing. To increase the experimental throughput, various next-generation sequencing (NGS)-based protocols have been developed, which generate libraries of pooled Ig heavy and light chain transcripts while preserving the heavy:light chain pairing of the individual cell [4–6]. We previously established a reverse-transcription PCR (RT-PCR) based methodology to analyze FC-sorted single murine or human B cells [5, 7]. In this process (matrix PCR), the Ig heavy and light chain transcripts are amplified in a nested PCR with two-dimensionally barcoded primer sets, which encode the physical location of the individual cell within a set of microtiter plates. The resulting library can be sequenced on Roche/454 or Illumina platforms. The indexed FC data recorded for each cell allows linkage of Ig sequence information to cell surface marker expression at single-cell level.

### Comparison to current ARR analysis tools

Automated data analysis pipelines are critical for high-throughput datasets since they facilitate fast and standardized analysis of ARR. Current analysis methods include online tools for analysis of antigen receptor sequences, which provide basic immunological sequence annotation, e.g. IMGT/HighV-QUEST [8]. Other more specialized computation pipelines like IGGalaxy [9], LymAnalyzer [10] or pRESTO [11] are used for analysis of antigen sequencing data. Change-O [12] additionally allows analysis of clonality, molecular evolution or somatic hypermutation. Although these computational methods have successfully been used to process and analyze bulk ARR sequencing data, none of them is designed to handle single-cell data. These tools do not provide integrated solutions to represent single cells in the form of data structures or identifiers and thus do not facilitate direct linkage of phenotypic data to single-cell sequences.

We developed sciReptor as a flexible single-cell ARR analysis toolkit. Its modular architecture allows analysis and comparison of Ig sequencing data originating from various experimental protocols. The core of sciReptor is constituted by a relational database, which stores all sequences, annotations and metadata in a standardized format. The usage of a relational database for data storage is distinct from other existing tools and allows sciReptor to link different data types such as sequences, FC data or genomic annotations of reference sequences. The parameters and reference sequences used by the analysis modules are customizable for individual projects.

## Implementation

**Fig. 1:**
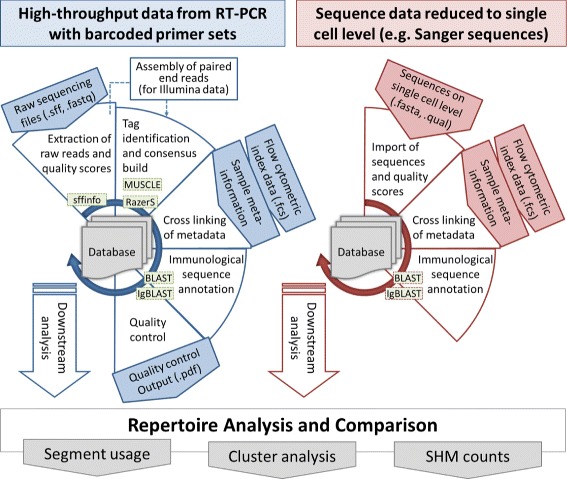
sciReptor analysis workflow. Overview of the sciReptor workflow for different datasets. *Blue*: processing steps for high-throughput data. *Red*: analysis workflow for data that is already on single-cell level. SHM: somatic hypermutations

sciReptor was initially developed to analyze sequencing data from our previously published single-cell matrix PCR platform [5], but the software is equally capable of processing data generated by alternative experimental procedures (Sanger sequencing or other NGS platforms). The latter requires that the sequencing reads have already been mapped to an individual cell and, if necessary, aggregated to provide a single consensus sequence per locus (Fig. 1). The algorithms for sequence annotation and metadata linkage are identical for different input data types. The required external software packages are given in Table 1. A step-by-step manual on how to process data is included in the repository, in addition, test data sets (Table 2) and a pre-configured virtual machine are available at http://b-cell-immunology.dkfz.de. Computationally expensive steps such as sequence alignment or consensus building can easily be parallelized on suitable hardware. The sequential steps of data input and sequence annotation are shown in Fig. 1: The blue panel (left) depicts processing of high-throughput sequencing data, whereas the red panel (right) side illustrates the analysis of Sanger sequences. The implementation details of consensus building from raw data and subsequent immunological and phenotypical annotation are described below.

**Table 1:** Required external software packages

Software	Version	Additional packages/comments
IgBLAST	1.4.0	
BLAST	2.2.30+	
Razers3	3.1.1./3.2 [13859]	
MUSCLE	3.8.31	
gsSeqTools	2.9 (build 20130529_1641)	optional for sff conversion
Perl	5.16.3	BioPerl (1.6.924)
R	3.1.1	BioConductor (2.26.0)
		FlowCore (1.32.0)
		RMySQL (0.9-3)
Python	2.7.5	numpy
		matplotlib
		MySQL-python
MariaDB	5.5.37	
git	1.8.3.1

**Table 2:** Human and murine single-cell test data sets from NGS and Sanger sequencing

Dataset	1	2	3
Number of sorted cells	1152	1152	384
Species	human	murine	murine
Experimental protocol	Matrix PCR	Matrix PCR	conventional PCR
Sequencing technique	Roche/454	Roche/454	Sanger
Published in	[7]	[5]	ENA Accession No
			LN879549–LN879837

### Input of raw sequence data and consensus building

When processing sequencing data from our matrix PCR platform, sciReptor maps each read to an individual cell and locus and subsequently builds an error-corrected consensus (Fig. 1). In detail, sciReptor first identifies the proximal and distal tag of each read with RazerS [13]. Then IgBLAST [14] and BLAST [15] are used to identify the most probable germline V, D and J segment and constant region for each read. All raw read information and statistics are stored in the database. The consensus build algorithm then successively identifies all reads originating from the same cell and locus and selects the reads with the most frequent V-J combination. MUSCLE [16] is used to align the reads and build a primary consensus, which is stored in the database. If possible, sciReptor will repeat this procedure to build a secondary consensus based on the reads with the second most common V-J combination, which facilitates the identification of multiple transcripts that were present in a single cell.

For sequencing data from other experimental procedures (as noted above), the tag identification and consensus build are skipped (Fig. 1). sciReptor then directly analyzes the single-cell level sequence data as provided by the user.

### Immunological sequence annotation

After successful assignment of sequences to single cells, IgBLAST is used for alignment versus a germline database and annotation of V, D and J segments or positions of functional Ig subregions (i.e. framework and complementarity determining regions). sciReptor provides its own algorithm for annotating SHM, which uses the query-to-germline alignment provided by IgBLAST and maps all mutations to the corresponding germline base pair. Isotypes are assigned for sequences containing sufficient parts of the constant region by using BLAST alignment versus a reference database.

### Integration of meta- and phenotypic data

To complete the information stored in the database, sciReptor links a set of user-defined specifications from a spreadsheet. This metadata includes information on the donor, the sample and the FC single-cell isolation process. Additionally, FC index data (including reagents and fluorochromes) can be linked to the sequence data.

### Relational database

The structure of the database reflects the different data types that are generated in the consecutive analysis steps of sciReptor. It is divided into four sections as depicted in Additional file 1: Figure S1: 1) data related to raw sequencing reads, 2) analysis results related to consensus sequences at single-cell level, 3) metadata and FC index data, 4) annotation of reference germline variable (V), diversity (D) and joining (J) segments and constant regions.

## Results and discussion

The advantage of sciReptor over currently available analysis pipelines is its capacity to handle single-cell data and directly assign it to other biological non-sequence data. To demonstrate this, we processed different B cell receptor datasets using sciReptor and visualized the single-cell linkage between heavy and light chain transcripts and cell surface marker expression. Table 2 shows the three available test datasets with their respective number of sorted single cells. The datasets are available at http://b-cell-immunology.dkfz.de. The analysis tools used to generate the graphical output are also part of the sciReptor software and can be accessed at the project’s repository https://github.com/b-cell-immunology.

### Quality control

sciReptor includes a quality control module for high-throughput sequencing datasets. The module visualizes the distribution of sequencing read lengths, the success rate of sequence tag identification and the distribution of reads per well. This allows the monitoring of sequencing depth as well as potential cross-contaminations. An example of the quality control output is shown in Additional file 1: Figures S2–S4.

### Linkage of heavy:light chain information

**Fig. 2:**
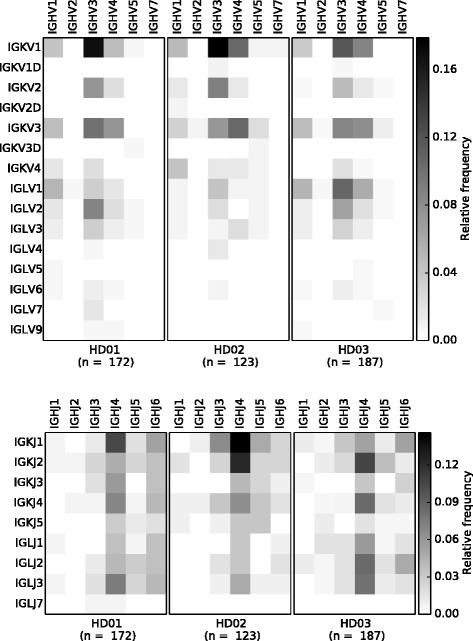
Paired Ig heavy and light chain segment usage. The analysis module of sciReptor comprises functions to plot the associations between heavy and light chain segments. The upper heatmaps represent relative association frequencies between heavy and light V segment families for each individual donor. The lower panel shows association frequencies of heavy and light J segment families

sciReptor attributes an event identifier (ID) to every cell included in the analysis. Every heavy, kappa or lambda sequence that is identified during data processing is then assigned to the corresponding event ID. Grouping the sequences by event ID provides information on the associated heavy and light chains of individual B cells. Figure 2 shows the association of Ig heavy and light chain V and J segments in three healthy human donors (dataset 1).

### Integration of flow cytometric index data

**Fig. 3:**
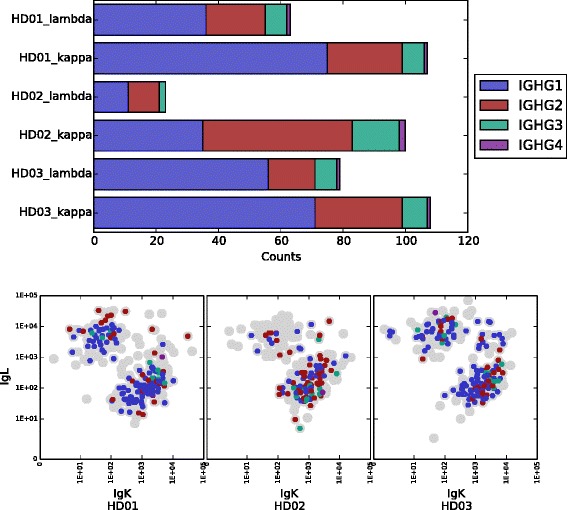
Isotype distribution related to flow cytometric index data. *Upper* panel: Distribution of IgG isotypes for all cells for which paired Ig heavy and light chain sequences could be determined. The data of each donor is split into two categories according to whether sciReptor identified an associated kappa or lambda chain. *Lower* panels: Indexed flow cytometry data of the sorted cells that were subjected to the sequencing process (gray dots). During the single-cell isolation, the cells were labeled with anti-Ig *κ* and anti-Ig *λ* antibodies (conjugated to PE-Cy7 and PE, respectively), whose respective fluorescence intensity is plotted. The cells for which Ig heavy and light chain sequences could be obtained are additionally color-coded according to the identified IgG isotype

In addition to linked single-cell heavy and light chain information, sciReptor also supports attribution of phenotypic characteristics. Figure 3 shows how sciReptor can be used to integrate single-cell sequence information with FC index data. The analysis is shown for IgG memory cells of three human donors (dataset 1). Sequences are grouped according to donor and usage of kappa or lambda chain. Different subclasses of IgG isotype are represented in the color scheme. The corresponding single cells are depicted in flow cytometric index plots showing their Ig *κ* and Ig *λ* surface expression.

sciReptor possesses the unique feature to handle single-cell Ig sequencing data. The generic structure of the database and algorithms are designed to be modular and can easily be adapted to handle T cell receptor (TCR) data as well. Additionally we are currently developing an analysis module to integrate antigen binding data generated with recombinantly expressed monoclonal antibodies.

## Conclusion

sciReptor is a flexible toolkit for the standardized analysis of single-cell Ig sequencing data. Its relational database backend allows integration of different types and sets of data and thus facilitates repertoire comparisons.

## Availability and requirements

**Project name:** sciReptor**Project homepage:**https://github.com/b-cell-immunology/sciReptorhttps://github.com/b-cell-immunology/sciReptor**Operating System:** Linux**Programming languages:** Perl, R, Python**Other requirements:** MariaDB, IgBLAST, BLAST, RazerS3, MUSCLE**License:** GNU Affero General Public License V3

## Additional file

Additional file 1
**Database structure and quality control.** Visual representation of the relational database used by sciReptor. Quality control output showing the read length distribution, the statistics for sequence tag identification and the reads per cell statistics. (PDF 369 kb)

## Abbreviations

ARR: Antigen receptor repertoire
FC: Flow cytometry
Ig: Immunoglobulin
NGS: Next-generation sequencing
PCR: Polymerase chain reaction
SHM: Somatic hypermutation
