# Supplementary material for: sciReptor: analysis of single-cell level immunoglobulin repertoires
Source: BMC Bioinformatics. 2016 Feb 4;17:67. doi: 10.1186/s12859-016-0920-1 (PMC4743164; doi:10.1186/s12859-016-0920-1)
Supplement: Additional file 1 — Database structure and quality control. Visual representation of the relational database used by sciReptor. Quality control output showing the read length distribution, the statistics for sequence tag identification and the reads per cell statistics. (PDF 369 kb) [file 12859_2016_920_MOESM1_ESM.pdf]

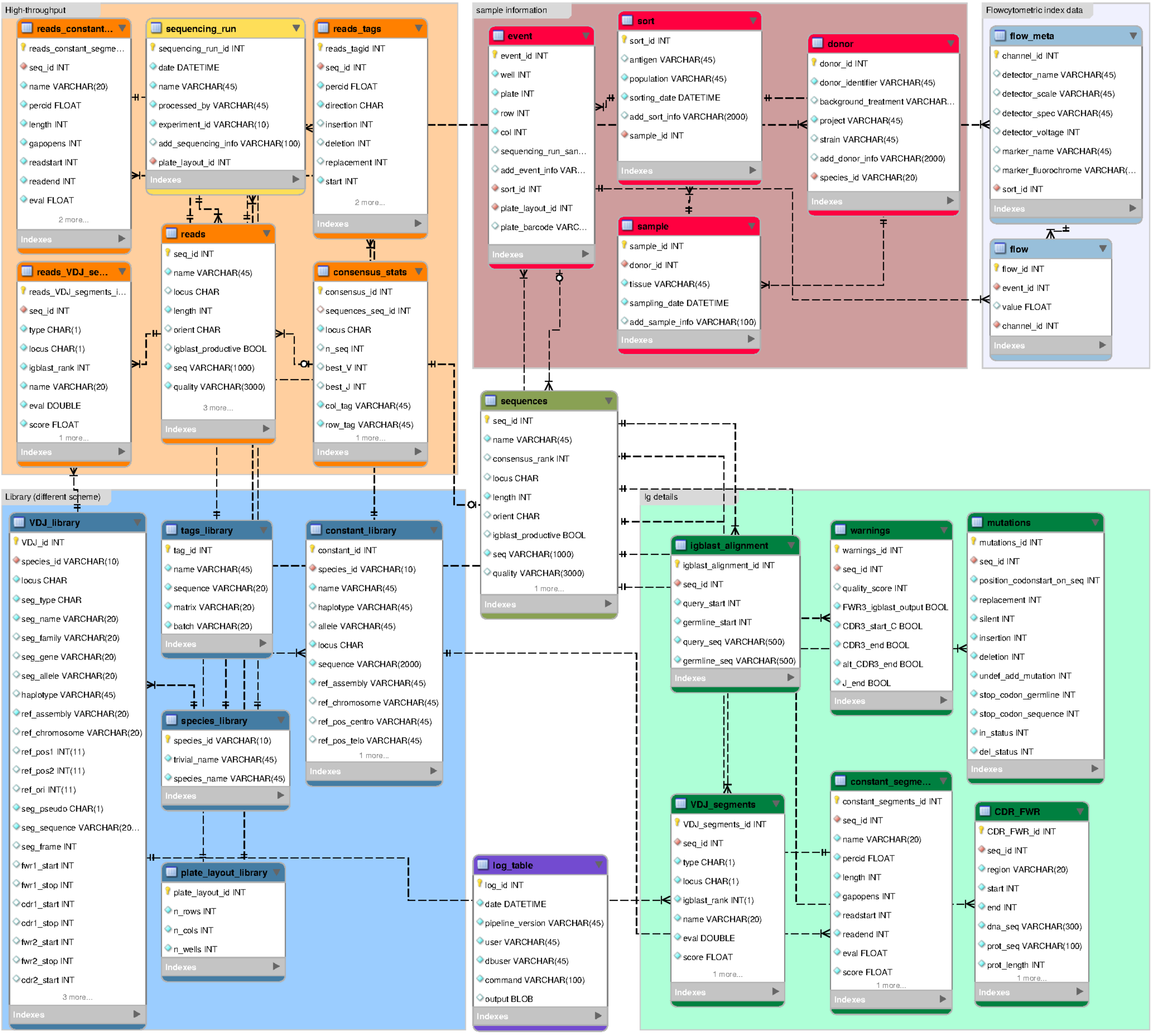

Figure 1: **sciReptor database scheme.** Orange: Data related to high-throughput matrix PCR (raw reads). Green: Data related to immunoglobulin sequences on single-cell level. Red: Metadata. Light blue: Indexed flow cytometry data. Dark blue: Reference germline V, D and J segments and constant regions including sequences and functional annotations. Purple: Logging. An interactive MySQL Workbench Model can be downloaded from `http://b-cell-immunology.dkfz.de/sciReptor_supplementary/sciReptor.mwb`.

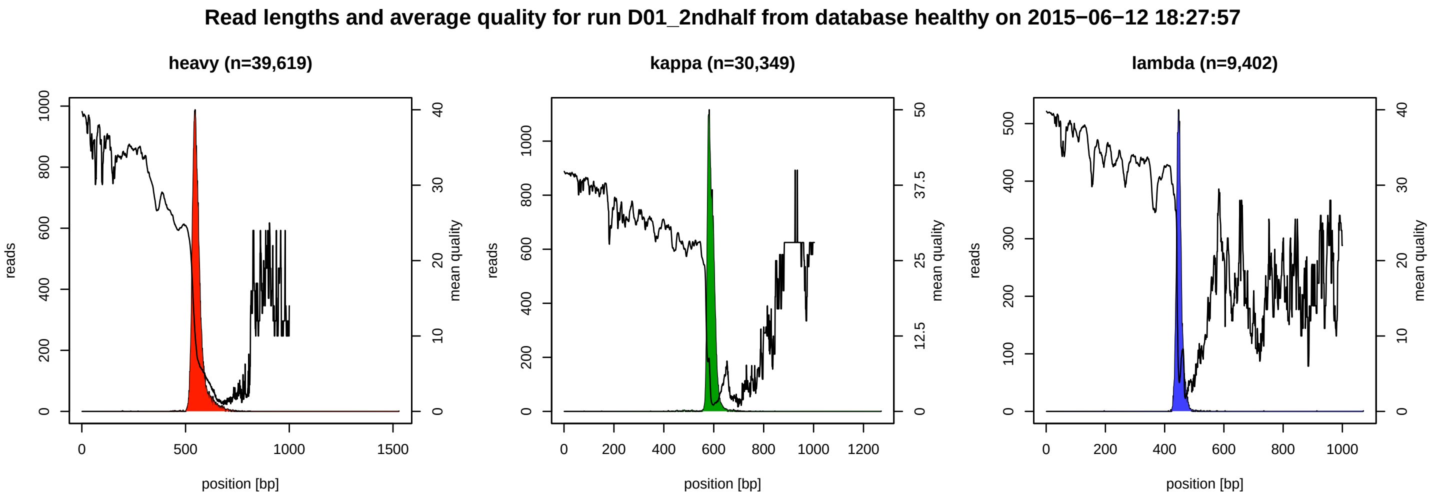

Figure 2: **Raw read sequence quality for human test dataset.** Sequence length distribution as well as mean sequence quality is shown for heavy, kappa and lambda chains separately. The average Phred score of raw reads is plotted as a function of the base position (black line, right axis of ordinates). A colored histogram shows the length distribution (left axis of ordinates). The observed length distribution is consistent with the expected amplicon size given the primers used (Murugan *et al.*, 2015).

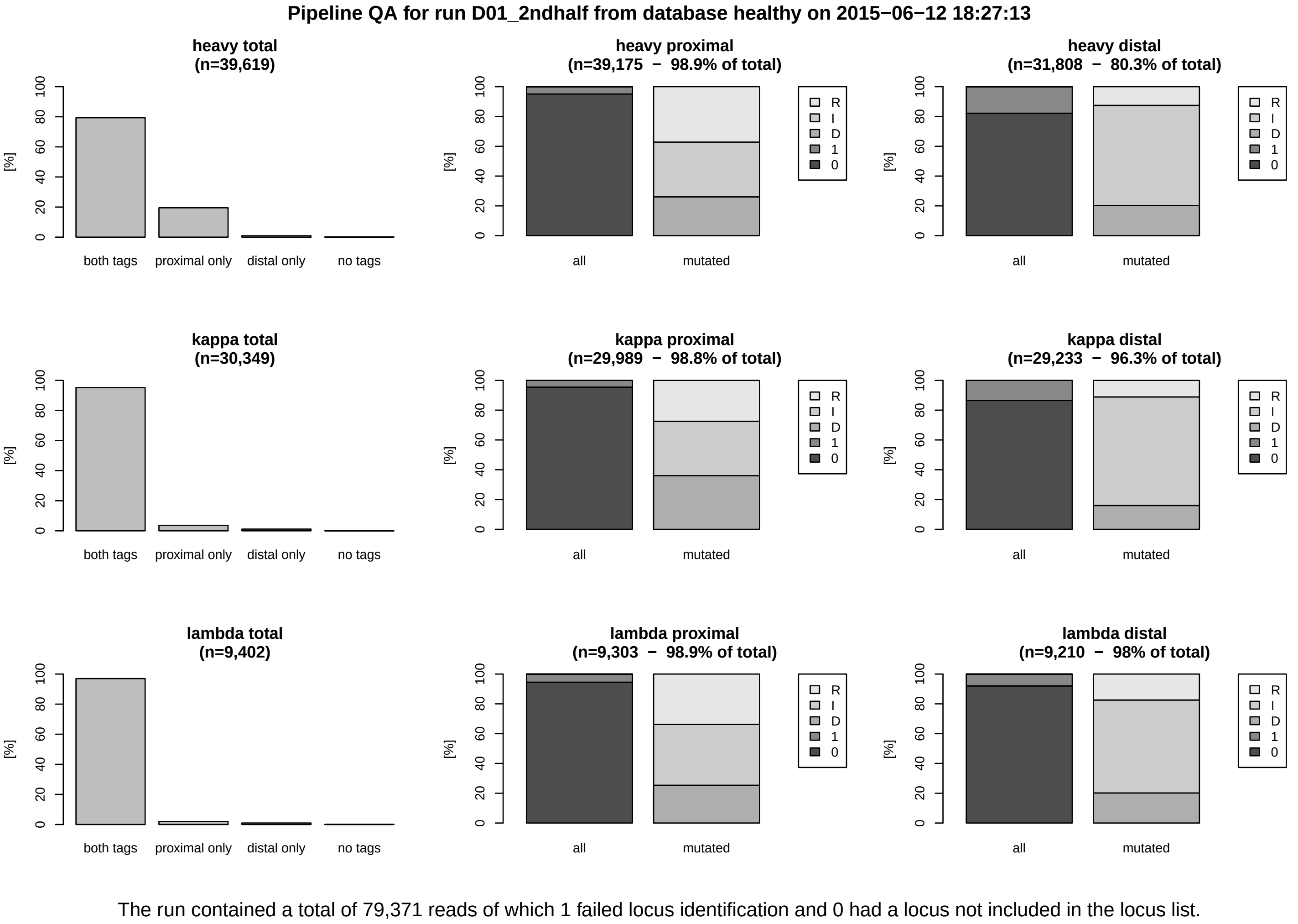

Figure 3: **Tag identification statistics.** In order to assign raw reads to a certain well of origin, sciReptor identifies proximal and distal tags. The left column represents the overall statistics of tag identification for reads mapped to heavy, kappa and lambda loci. The middle and right columns show a more detailed view of proximal and distal tag identification, respectively. The percentage of tags with one mismatch (denoted as '1' in the legend) among the overall identified tags is shown in the left stacked bargraph of each panel ('all'). The distribution of nucleotide replacement (R), insertion (I) and deletion (D) mutations is shown in the right bargraph ('mutated').

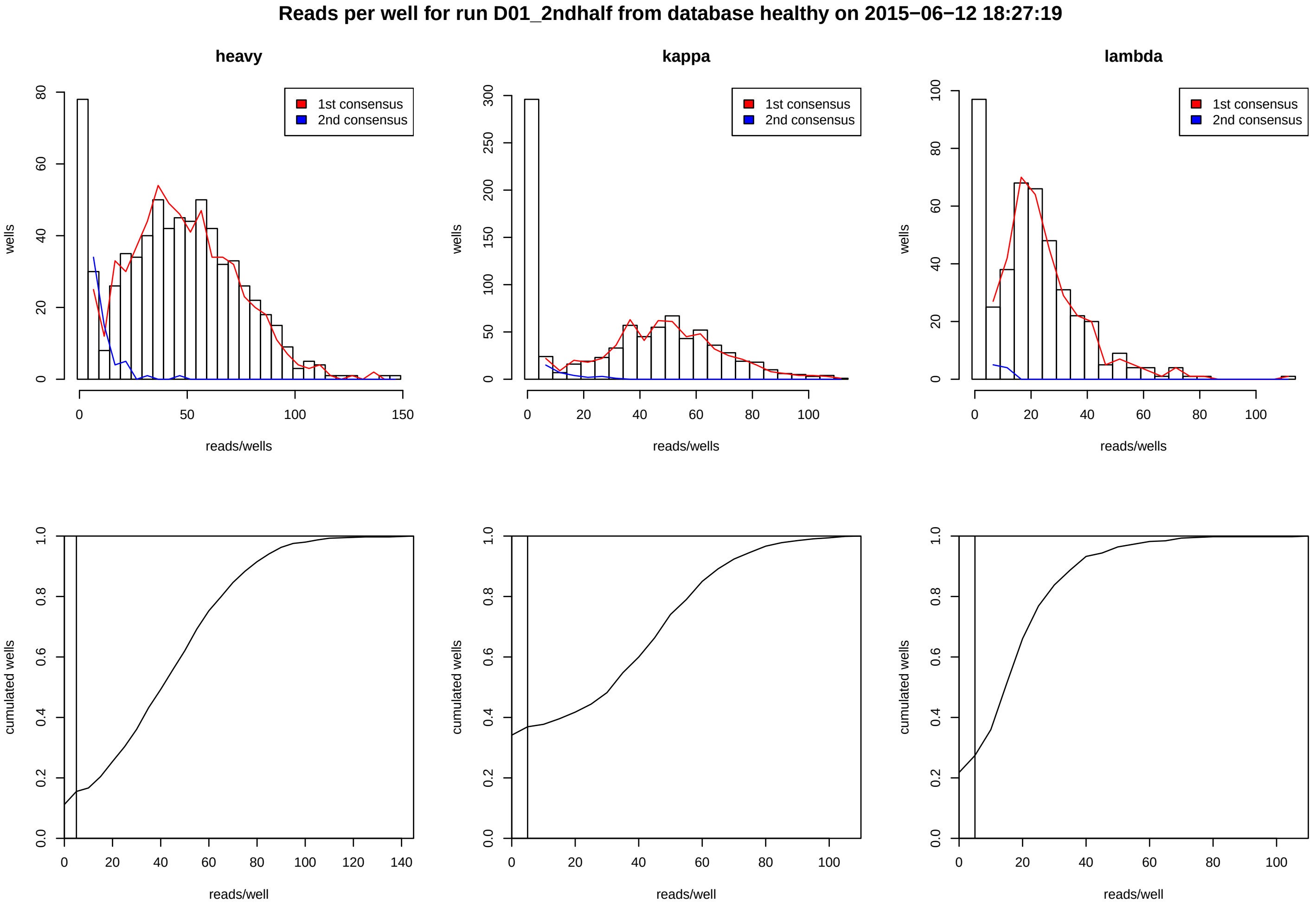

Figure 4: **Read mapping to single wells.** The reads per well distribution for heavy, kappa and lambda sequences is shown in the upper panels. Refer to the main text for definition of first and second consensus. The lower panels illustrate the respective cumulative read distribution for first consensi. The vertical line at x = 5 illustrates the cutoff of minimum five reads per well to build a consensus sequence.
